# Cardiovascular disease in women: insights from magnetic resonance imaging

**DOI:** 10.1186/s12968-020-00666-4

**Published:** 2020-09-28

**Authors:** Chiara Bucciarelli-Ducci, Ellen Ostenfeld, Lauren A. Baldassarre, Vanessa M. Ferreira, Luba Frank, Kimberly Kallianos, Subha V. Raman, Monvadi B. Srichai, Elisa McAlindon, Sophie Mavrogeni, Ntobeko A. B. Ntusi, Jeanette Schulz-Menger, Anne Marie Valente, Karen G. Ordovas

**Affiliations:** 1 Bristol Heart Institute, Bristol National Institute of Health Research (NIHR) Biomedical Research Centre, University Hospitals Bristol and University of Bristol, Bristol, UK; 2 Department of Clinical Sciences Lund, Clinical Physiology, Skåne University Hospital Lund, Lund University, Getingevägen 5, SE-22185 Lund, Sweden; 3 Yale University, New Haven, CT USA; 4 Oxford Centre for Clinical Magnetic Resonance Research (OCMR), Division of Cardiovascular Medicine, British Heart Foundation Centre of Research Excellence, Oxford NIHR Biomedical Research Centre, University of Oxford, Oxford, UK; 5 University of Texas Medical Branch, Galveston, TX USA; 6 University of California San Francisco, San Francisco, CA USA; 7 Ohio State University, Columbus, OH USA; 8 Georgetown University, Washington DC, USA; 9 Heart and Lung Centre, New Cross Hospital, Wolverhampton, UK; 10 Onassis Cardiac Surgery Center, Athens, Greece; 11 University of Cape Town and Groote Schuur Hospital, Cape Town, South Africa; 12 Charite Hospital, University of Berlin and HELIOS-Clinics Berlin-Buch, Berlin, Germany; 13 Boston Children’s Hospital, Brigham and Women’s Hospital, Boston, USA

**Keywords:** Cardiovascular magnetic resonance, Female cardiovascular disease, Ischemic heart disease, Non-ischemic cardiomyopathies, Peripartum cardiomyopathy, Chemotherapy-induced cardiomyopathy, Congenital heart disease, Turner syndrome, Connective tissue disease, Pulmonary hypertension

## Abstract

The presentation and identification of cardiovascular disease in women pose unique diagnostic challenges compared to men, and underrecognized conditions in this patient population may lead to clinical mismanagement.

This article reviews the sex differences in cardiovascular disease, explores the diagnostic and prognostic role of cardiovascular magnetic resonance (CMR) in the spectrum of cardiovascular disorders in women, and proposes the added value of CMR compared to other imaging modalities. In addition, this article specifically reviews the role of CMR in cardiovascular diseases occurring more frequently or exclusively in female patients, including Takotsubo cardiomyopathy, connective tissue disorders, primary pulmonary arterial hypertension and peripartum cardiomyopathy. Gaps in knowledge and opportunities for further investigation of sex-specific cardiovascular differences by CMR are also highlighted.

## Background

Women are commonly underrepresented in cardiovascular research, comprising as little as 15–35% of populations in randomized clinical trials [1], and when included, are most often in the postmenopausal stage of life [2]. Even though both men and women are affected by cardiovascular disease, there are only limited numbers of sex-specific and age-balanced imaging and management guidelines for women with cardiovascular disease [2]. In the setting of growing awareness of providing personalized precision medicine, addressing sex differences in cardiovascular disease is a key goal [1].

Anatomically, women have smaller hearts even after adjustment for body size, and, as a result, have different disease phenotypes, which may influence the choice and accuracy of diagnostic tests. Further, there are intrinsic imaging difficulties with transthoracic echocardiography in women due to reduced image quality from breast tissue attenuation and reluctance to use cardiac computed tomography (CCT) in pre-menopausal women due to breast tissue sensitivity. Cardiovascular magnetic resonance (CMR) imaging provides a comprehensive evaluation of cardiovascular disease, including assessment of myocardial structure and function, inflammation, ischemia, viability, and valvular disease, with the additional benefit of excellent reproducibility [3]. The advantage of lack of exposure to ionizing radiation is particularly beneficial in women, especially in those of childbearing and premenopausal age.

The literature on sex-specific differences in cardiovascular conditions, including sex-specific CMR reference values, is limited. The aims of this paper are to review the applications of CMR in the spectrum of cardiovascular diseases that affect women, with a particular focus on sex-related differences in conditions that occur more frequently or exclusively in women.

## Comparison of normative CMR sex-specific values in healthy subjects

Table 1 lists normative values of ventricular volumes and mass in women and men.

**Table 1:** Normative CMR values of cardiac volumes and function in women and men

	Author, year	N (women + men), age	Women	Men	Women compared to men
**Left ventricle**					
LVEDV (ml)	Petersen et al., 2017 [4]	433 + 371, 45–74 years	124 (88–161)	166 (109–218)	**↓**
	Maicera et al., 2006 [5]	60 + 60, 20–80 years	128 (88–168)	156 (115–198)	**↓**
	Alfakih et al., 2003 [6]	30 + 30, 20–65 years	135 (96–174)	169 (102–235)	**↓**
LVEDVi (ml/m^2^)	Petersen et al., 2017 [4]	433 + 371, 45–74 years	74 (54–94)	85 (60–110)	**↓**
	Maicera et al., 2006 [5]	60 + 60, 20–80 years	75 (57–92)	80 (63–98)	**↓**
	Alfakih et al., 2003 [6]	30 + 30, 20–65 years	78 (56–99)	82 (53–112)	**↓**
LVESV (ml)	Petersen et al., 2017 [4]	433 + 371, 45–74 years	49 (31–68)	69 (39–97)	**↓**
	Maceira et al., 2006 [5]	60 + 60, 20–80 years	42 (23–60)	53 (30–75)	**↓**
	Alfakih et al., 2003 [6]	30 + 30, 20–65 years	49	61	**↓**
LVESVI (ml/m^2^)	Petersen et al., 2017 [4]	433 + 371, 45–74 years	29 (19–40)	36 (21–49)	**↓**
	Maceira et al., 2006 [5]	60 + 60, 20–80 years	24 (15–34)	27 (16–38)	**↓**
LVSV (ml)	Petersen et al., 2017 [4]	433 + 371, 45–74 years	75 (49–100)	96 (59–132)	**↓**
	Maceira et al., 2006 [5]	60 + 60, 20–80 years	86 (58–114)	104 (76–132)	**↓**
	Alfakih et al., 2003 [6]	30 + 30, 20–65 years	86	108	**↓**
LVSVI(ml/m^2^)	Petersen et al., 2017 [4]	433 + 371, 45–74 years	45 (30–59)	49 (32–67)	**↓**
	Maceira et al., 2006 [5]	60 + 60, 20–80 years	50 (38–63)	53 (41–65)	**↓**
LVEF (%)	Petersen et al., 2017 [4]	433 + 371, 45–74 years	61 (51–70)	58 (48–69)	**↑**
	Maceira et al., 2006 [5]	60 + 60, 20–80 years	67 (58–76)	67 (58–75)	**→**
	Alfakih et al., 2003 [6]	30 + 30, 20–65 years	64 (54–74)	64 (55–73)	**→**
LVM (g)	Petersen et al., 2017 [4]	433 + 371, 45–74 years	70 (46–93)	103 (64–141)	**↓**
	Maceira et al., 2006 [5]	60 + 60, 20–80 years	108 (72–144)	146 (108–184)	**↓**
	Alfakih et al., 2003 [6]	30 + 30, 20–65 years	90 (66–114)	133 (85–181)	**↓**
LVMI (g/m^2^)	Petersen et al., 2017 [4]	433 + 371, 45–74 years	42 (29–55)	53 (35–70)	**↓**
	Maceira et al., 2006 [5]	60 + 60, 20–80 years	63 (48–77)	74 (58–91)	**↓**
	Alfakih et al., 2003 [6]	30 + 30, 20–65 years	52 (37–67)	65 (46–83)	**↓**
**Right ventricle**					
RVEDV (ml)	Petersen et al., 2017 [4]	433 + 371, 45–74 years	130 (85–168)	182 (124–258)	**↓**
	Maceira et al., 2006 [7]	60 + 60, 20–80 years	126 (84–168)	163 (113–213)	**↓**
	Alfakih et al., 2003 [6]	30 + 30, 20–65 years	131 (83–178)	177 (111–243)	**↓**
RVEDVI (ml/m^2^)	Petersen et al., 2017 [4]	433 + 371, 45–74 years	77 (53–99)	93 (68–125)	**↓**
	Maceira et al., 2006 [7]	60 + 60, 20–80 years	73 (55–92)	83 (60–106)	**↓**
	Alfakih et al., 2003 [6]	30 + 30, 20–65 years	75 (48–103)	86 (58–114)	**↓**
RVESV (ml)	Petersen et al., 2017 [4]	433 + 371, 45–74 years	55 (27–77)	85 (47–123)	**↓**
	Maceira et al., 2006 [7]	60 + 60, 20–80 years	43 (17–69)	57 (27–86)	**↓**
	Alfakih et al., 2003 [6]	30 + 30, 20–65 years	52	79	**↓**
RVESVI (ml/m^2^)	Petersen et al., 2017 [4]	433 + 371, 45–74 years	33 (17–46)	43 (25–63)	**↓**
	Maceira et al., 2006 [7]	60 + 60, 20–80 years	25 (12–38)	29 (14–43)	**↓**
RVSV (ml)	Petersen et al., 2017 [4]	433 + 371, 45–74 years	75 (48–99)	97 (68–125)	**↓**
	Maceira et al., 2006 [7]	60 + 60, 20–80 years	83 (57–108)	106 (72–140)	**↓**
	Alfakih et al., 2003 [6]	30 + 30, 20–65 years	78	98	**↓**
RVSVi (ml/m^2^)	Petersen et al., 2017 [4]	433 + 371, 45–74 years	45 (30–59)	50 (34–67)	**↓**
	Maceira et al., 2006 [7]	60 + 60, 20–80 years	48 (36–60)	54 (38–70)	**↓**
RVEF (%)	Petersen et al., 2017 [4]	433 + 371, 45–74 years	58 (47–68)	54 (45–65)	**↑**
	Maceira et al., 2006 [7]	60 + 60, 20–80 years	66 (54–78)	66 (53–78)	**→**
	Alfakih et al., 2003 [6]	30 + 30, 20–65 years	60 (50–70)	55 (48–63)	**↑**
RVM (g)	Maceira et al., 2006 [7]	60 + 60, 20–80 years	48 (27–69)	66 (38–94)	**↓**
RVMi (g/m^2^)	Maceira et al., 2006 [7]	60 + 60, 20–80 years	28 (18–38)	34 (19–43)	**↓**
**Left atrium**					
LAV max (ml)	Petersen et al., 2017 [4]^a^	433 + 371, 45–74 years	62 (33–93)	71 (37–108)	**↓**
	Maceira et al., 2010 [8]^c^	60 + 60, 20–80 years	68 (42–95)	77 (48–107)	**↓**
LAVi max (ml/m^2^)	Petersen et al., 2017 [4]^a^	433 + 371, 45–74 years	37 (21–55)	36 (19–55)	**→**
	Maceira et al., 2010 [8]^c^	60 + 60, 20–80 years	40 (27–52)	39 (26–53)	**→**
LA emptying fraction (%)	Petersen et al., 2017 [4]^a^	433 + 371, 45–74 years	61 (49–74)	60 (47–73)	**→**
	Maceira et al., 2016 [9]^c^	60 + 60, 20–80 years	60 (48–72)	58 (47–68)	**↑**
**Right atrium**					
RAV max (ml)	Petersen et al., 2017 [4]^b^	433 + 371, 45–74 years	69 (38–101)	93 (43–143)	**↓**
	Maceira et al., 2013 [10]^c^	60 + 60, 20–80 years	91 (58–124)	109 (64–124)	**↓**
RAVi max (ml/m^2^)	Petersen et al., 2017 [4]^b^	433 + 371, 45–74 years	41 (23–59)	48 (22–74)	**↓**
	Maceira et al., 2013 [10]^c^	60 + 60, 20–80 years	53 (36–70)	55 (33–78)	**→**
RA emptying fraction (%)	Petersen et al., 2017 [4]^b^	433 + 371, 45–74 years	46 (31–63)	41 (23–58)	**↑**
	Maceira et al., 2016 [9]^c^	60 + 60, 20–80 years	58 (46–69)	54 (40–68)	**↑**

Data expressed as mean and in parenthesis the lower and upper reference limits (95% interval) when noted in original publication

*LV* Left ventricular, *EDV* End-diastolic volume, *I* Indexed to body surface area, *ESV* End-systolic volume, *EF* Ejection fraction, *LVM* Left ventricular mass, *RV* Right ventricular, *LAV* Left atrial volume, *LA* Left atrial, *RAV* Right atrial volume, *RA* Right atrial

^a^Volumes from biplane, ^b^ Volumes from single plane 4ch view, ^c^ Volumes from short axis stack

Both absolute and indexed left ventricular (LV) and right ventricular (RV) volumes and LV mass are smaller in women compared to men. LV and RV ejection fraction (EF) are greater or equal in women compared to men [4–7, 11]. Absolute left atrial (LA) maximal volume is significantly smaller in women compared to men, however indexed LA volumes and emptying fraction are similar between sexes [4, 8, 9]. Absolute right atrial (RA) maximal volume is significantly smaller, indexed RA volume is smaller or equal, and RA emptying fraction is higher in women [4, 9, 10].

Current evidence suggests that T1 and extracellular volume (ECV) values are higher in women, especially premenopausal women, compared with men, at both 1.5 T and 3 T [12–15]. There is conflicting evidence whether CMR T2 mapping values are influenced by sex [15–18], although current studies used different T2-mapping techniques, and may be underpowered to detect sex-dependent effects. Large population based studies that include equal sex representation will allow for sex-specific reference values for T1, T2 and ECV [19].

## Ischemic heart disease

### Acute myocardial infarction

Acute myocardial infarction (MI) in women differs from men in presentation, underlying pathophysiology, and outcomes [20, 21]. This includes a higher mortality after acute MI in women < 50 years of age (odds ratio 1.37 for female sex) [22]. Women have a higher prevalence of non-obstructive coronary plaques [21, 23–25] and less atheroma volume than men [26], which may affect strategies for diagnosing acute coronary syndrome (ACS) in women.

CMR can characterize myocardial tissue following MI, independently of the presence of obstructive coronary lesions. Studies have shown that infarct size and myocardial salvage are smaller in women than men (myocardial salvage index: women 0.4 vs. men 0.5, *p* = 0.013), reflecting a smaller acute infarct size (women 14% of LV vs. men 22% of LV) and follow up infarct size (women 8% vs. men 13% LV) [27]. In addition, microvascular obstruction (MVO) burden has been shown to be smaller in women than in men by Canali et al. (women 1.1 ± 1.0% LV vs. men 3.4 ± 2.2% LV, *p* = < 0.001) [27] and by Langerhans et al. (women 0.48 ± 1.3% LV vs. men 1.2 ± 3.0% LV, *p* = 0.03) [28].

#### Myocardial infarction with non-obstructed coronary arteries (MINOCA)

There is over-representation of women with MI with non-obstructed coronary arteries (MINOCA) relative to those with elevated troponin and obstructive coronary artery disease (CAD) (24–30% are women) [29, 30]. Mechanisms of MINOCA more commonly observed in women include coronary microvascular dysfunction [31–33] and coronary artery plaque erosions [34, 35]. Identification of underlying etiology of MINOCA is important for risk stratification and treatment decision-making [36, 37]. CMR is a key diagnostic imaging tool in the assessment of patients with MINOCA, providing detailed myocardial tissue characterization, location of myocardial inflammation/edema, scarring/fibrosis, and discriminating between ischemic and non-ischemic etiologies. CMR has been shown to identify the underlying etiology in up to 87% of patients with MINOCA [38]. In particular, the more common causes of MINOCA, such as myocarditis, acute MI without obstructing plaque, and Takotsubo cardiomyopathy (TCM), can be easily diagnosed with CMR. Ischemic patterns of late gadolinium enhancement (LGE) may be seen in women presenting with MINOCA [39], and abnormal perfusion on stress CMR is commonly noted, likely to be related to multiple mechanisms, including microvascular dysfunction [40]. Despite the absence of angiographically significant CAD, patients with MINOCA have worse outcomes with a 12 month all-cause mortality rate of 4.7% [29]. A recent study demonstrates that CMR can inform prognosis in MINOCA patients, independent of sex [41].

#### Differential diagnosis of MINOCA

##### Myocarditis

**Fig. 1:**
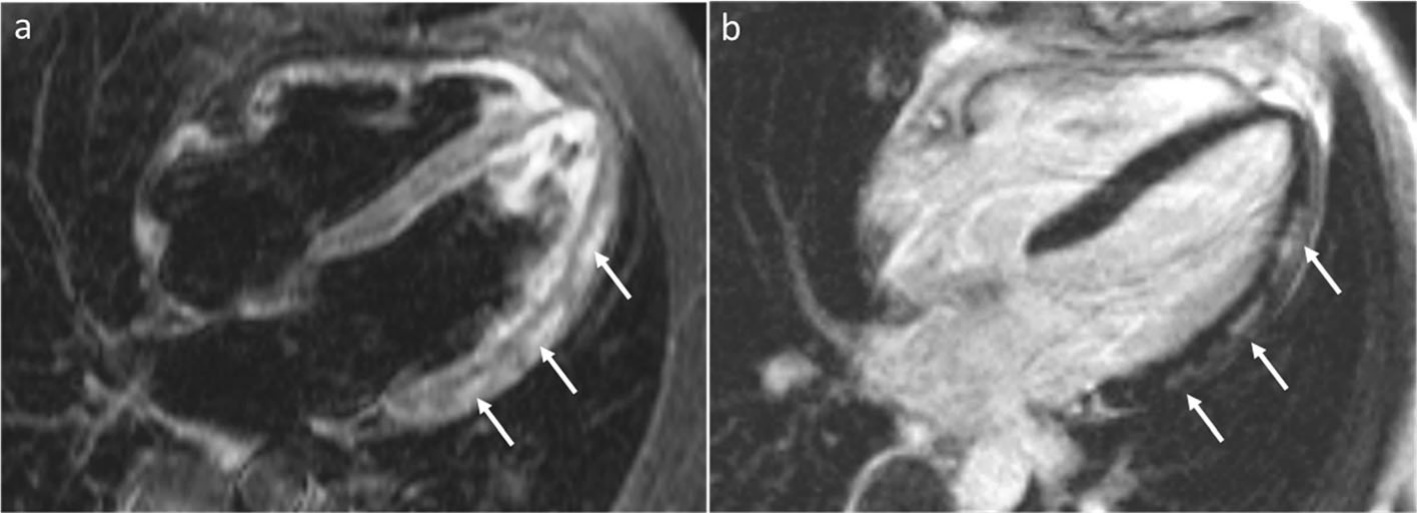
Acute Myocarditis. Four chamber long-axis view T2-weighted image (**a**) and corresponding late gadolinium enhancement (LGE) image (**b**). The white arrows indicate patchy epicardial and mid-wall areas of myocardial edema **a** with corresponding epicardial and mid-wall late enhancement (**b**)

CMR is an important tool for diagnosis of myocarditis in both sexes [42, 43]. The CMR diagnosis of myocarditis is based on the “Lake Louise criteria” of myocardial edema, hyperemia, and fibrosis (Fig. 1) [44]. In addition, parametric mapping techniques, including native T1 mapping, extracellular volumes of distribution, and T2 mapping are promising techniques and may significantly improve the diagnostic accuracy of CMR [14, 45–48].

Although there are no differences in the CMR diagnostic criteria for myocarditis in women versus men, sex-differences are noted specifically related to the subsequent risk of chronic dilated cardiomyopathy [49].

##### Takotsubo cardiomyopathy

**Fig. 2:**
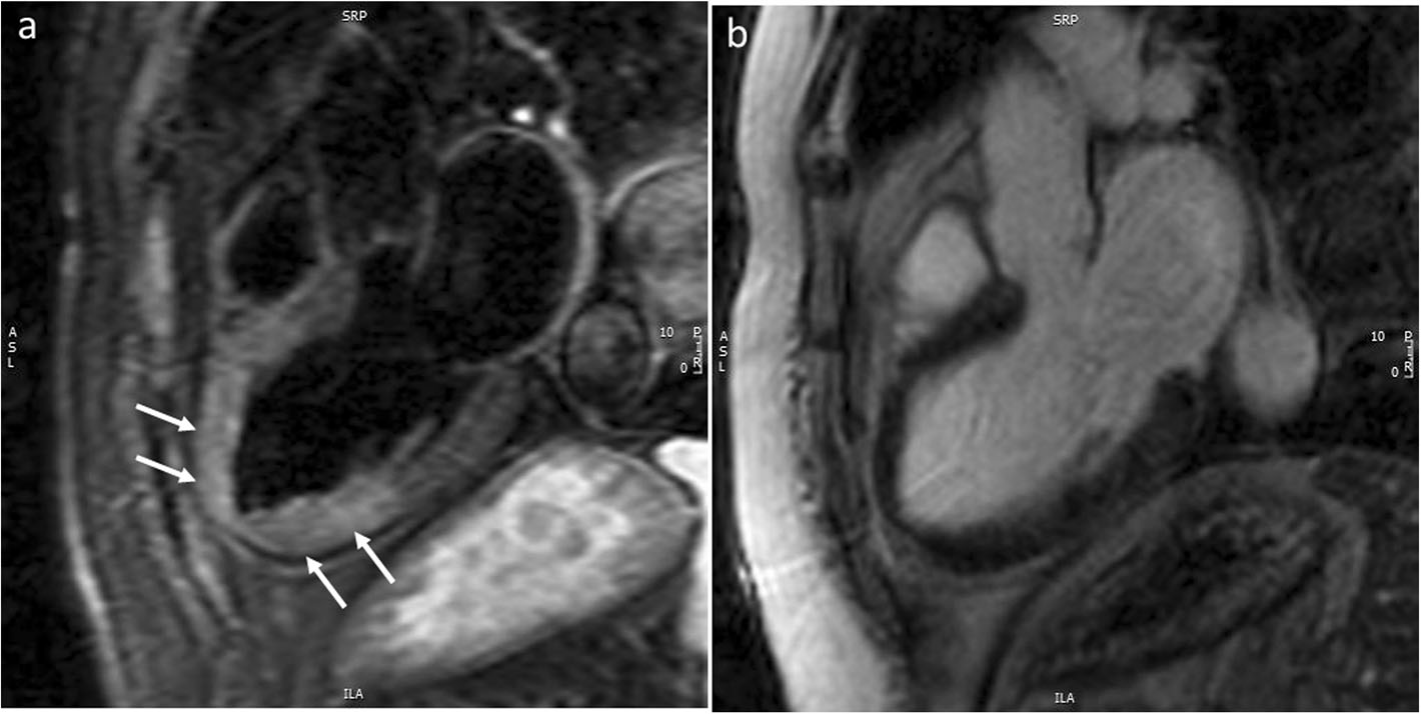
Takotsubo cardiomyopathy. Three-chamber view of a patient with Takotsubo cardiomyopathy. **a** shows the T2 weighted image with increased signal intensity of the mid-cavity and apical segments (white arrows) without late gadolinium enhancement (**b**)

Takotsubo cardiomyopathy (TCM) should be considered in the differential diagnosis of MINOCA, with a prevalence of 10–27% [36, 38, 50–52]. TCM is a condition more prevalent in women and is often precipitated by an emotional or physical stress and a characteristic finding is mid-cavity to apical akinesia with sparing of the basal segments though many atypical variants have been described. While previously thought to have a favorable prognosis, recent data suggest that TCM is associated with increased arrhythmic risk and worse prognosis [53, 54]. CMR has added diagnostic value in TCM, detecting myocardial edema in regions with focal wall motion abnormalities, without the presence of myocardial scarring (Fig. 2) [55]. The typical pattern of myocardial edema is circumferential, transmural in extent, and resolves within 2–3 months along with recovery of regional wall motion abnormality [56]. In TCM, absence of LGE rules out acute MI or myocarditis; although a subtle patchy LGE may be present, which has been attributed to the presence of edema [51, 56], sub-microscopic cell death, and transient increase in levels of extracellular matrix proteins, particularly collagen-1 [57].

#### Chronic coronary syndrome

The diagnosis of chronic coronary syndrome (previously referred to as stable CAD) presents several challenges in women partially due to a more atypical presentation and lower prevalence in women compared to men [58]. Women have a higher prevalence of angina [59] but lower prevalence of atherosclerosis and obstructive CAD, despite presenting at an older age and with a greater risk factor burden than men [22, 60]. The greater prevalence of non-obstructive CAD in women challenges the traditional diagnostic goal of detecting obstructive CAD needing revascularization and shifts the diagnostic focus to detecting ischemia. The Women’s Ischemia Syndrome Evaluation (WISE) study demonstrated that, even in the absence of obstructive coronary atherosclerosis, many women who present with chest pain have evidence of exercise-induced myocardial ischemia and coronary vasomotor dysfunction posing diagnostic challenges [61, 62].

The role of non-invasive imaging modalities available to evaluate women with stable ischemic heart disease has been illustrated [63]. The intrinsic advantages of CMR versus other methods are the ability to overcome the technical limitations of conventional stress imaging modalities, such as breast tissue, obesity, lung disease, and patients’ poor exercise capacity.

CMR studies in women with signs and symptoms of ischemia with or without obstructive CAD have made a number of observations, including the presence of increased native T1 values compared to controls, which was associated with reduced myocardial perfusion reserve index (MPRI), a potential surrogate measure of ischemia severity [64].

Stress CMR for detection of ischemia has proven to be an effective and robust risk stratification tool in patients of both sexes presenting with suspected CAD [65]. The CMR for Myocardial Perfusion Assessment in Coronary Artery Disease Trial (MR-IMPACT 2) was the first study demonstrating better diagnostic performance of stress CMR vs. single-photon emission computed tomography (SPECT) in certain populations, such as women [66]. The Magnetic Resonance Perfusion or Fractional Flow Reserve (FFR) in Coronary Artery Disease trial (MR-INFORM) showed that in patients with stable angina and risk factors for coronary artery disease, stress CMR was associated with a lower incidence of coronary revascularization than FFR and was noninferior to FFR with respect to major adverse cardiac events [67]. The study included only 28% women and sex-difference outcomes were not reported. The Clinical Evaluation of Magnetic Resonance Imaging in Coronary Heart Disease (CE-MARC) study demonstrated that the accuracy of SPECT was significantly worse in women than in men (*P* < 0.0001), whereas stress CMR outperformed SPECT in both women (area under the curve [AUC], 0.90 vs. 0.67) and in men (AUC, 0.89 vs. 0.74) [68]. In addition, women with false positive nuclear stress testing results who have a negative dobutamine stress CMR have a low likelihood of major adverse cardiovascular events [69].

While traditional non-invasive imaging tests are often normal in coronary microvascular dysfunction, stress CMR presents a diagnostic opportunity as highlighted by two studies. Painting et al. [70] showed that in patients with Syndrome X, semi-quantitative stress CMR could demonstrate subendocardial hypoperfusion compared to controls. Thomson et al. confirmed these findings in a larger cohort of patients with microvascular dysfunction confirmed by coronary reactivity testing [71].

Based on the available evidence, a sex-based diagnostic work up in ischemic heart disease by using CMR and CCT has been recently proposed [58, 72]. The American Heart Association (AHA) consensus statement on the role of non-invasive testing in the clinical evaluation of women with suspected ischemic heart disease [73] recommends CMR in symptomatic women with intermediate risk of CAD and resting ST-segment abnormalities or inability to exercise. In premenopausal women with functional disability, stress CMR may be reasonable for the identification of obstructive CAD and estimation of prognosis [73].

## Non-ischemic cardiomyopathies

### Peripartum cardiomyopathy

Peripartum Cardiomyopathy (PPCM) is defined as an idiopathic cardiomyopathy manifesting as heart failure due to LV systolic dysfunction in the final weeks of pregnancy or in the first 6 months after delivery when no other cause of heart failure is found [74]. The incidence of PPCM is highly variable among geographic regions, reported as 0.1% of pregnancies, but with high morbidity and mortality rates ranging 7 to 50% [75, 76]. Cardiovascular adaptive changes occur normally during pregnancy [77], and there are published reference CMR values for cardiac indices during pregnancy and the postpartum period in healthy pregnant women aged 18 to 35 years [77]. Typically, there is an increased left ventricular end-diastolic volume (LVEDV) and increased LV mass (LVM) during pregnancy, with these values consistently underestimated by echocardiography.

**Fig. 3:**
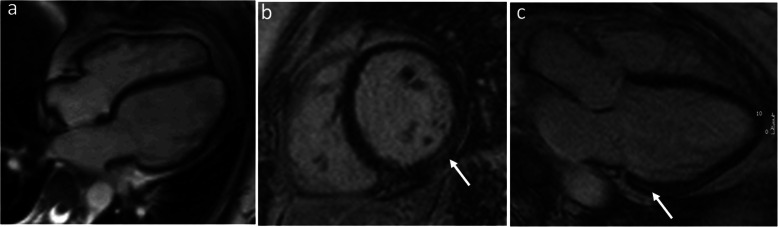
Peripartum cardiomyopathy. Cine long-axis four chamber view, end-diastolic frame (**a**), late gadolinium enhancement short-axis (**b**), and three chamber view (**c**) in a woman with postpartum cardiomyopathy. The images show only a mildly dilated LV cavity (**a**) and mid-wall late gadolinium enhancement of the basal inferolateral wall (white arrows)

While the initial imaging diagnosis of PPCM is based on echocardiography, CMR has a significant added value by accurately assessing LVEF and identifying myocardial edema and LGE [78, 79]. The mid-wall and subepicardial LGE pattern observed in PPCM can be seen in up to 40% of patients in the acute phase or in the follow up examinations. The presence and extent of LGE in PPCM has been linked to an unfavorable prognosis with slower recovery, higher risk of prolonged or permanent systolic dysfunction, and higher rate of developing heart failure exacerbation in future pregnancies (Fig. 3) [78, 79].

RV dysfunction evaluated serially by CMR has emerged as a negative prognostic indicator in patients with PPCM, as it is associated with increased dilation of both ventricles and lower LVEF, suggestive of more extensive biventricular cardiac involvement [78, 80].

### Breast cancer related chemotherapy-induced cardiomyopathy

Current therapy for breast cancer with anthracyclines and trastuzumab has resulted in significantly improved survival in women; however, it is associated with increased cardiovascular events, with over 7 times increased risk of heart failure and cardiomyopathy compared to patients who were not treated with chemotherapy [81]. Therefore, cardiac monitoring of women undergoing treatment for breast cancer is of extreme importance, as cardiovascular disease is now the leading cause of death in these survivors, accounting for 15.9% of deaths in one study [82].

Transthoracic echocardiography (TTE) is the first line imaging modality to screen and monitor cardiac function in breast cancer patients undergoing anti-cancer treatment [83]. However, CMR plays a growing role in this field [84]. Current guidelines offer recommendation for administration of potentially cardio-toxic chemotherapy based on LVEF assessment, with a decrease in the LVEF of as little as 10% prompting consideration of withholding therapy in some cases [85]. Therefore, the precise LVEF assessment provided by CMR is of utmost importance in cancer patients in need of cardiotoxic chemotherapy. In addition, a study of patients exposed to anthracyclines has demonstrated that, compared with CMR, 2D echo and 3D TTE had a false-negative rate of 75 and 47%, respectively, for detection of LVEF less than 50% [86]. Finally, TTE examination is often not well tolerated in post-surgical breast cancer patients due to significant discomfort at the post-surgical site. The use of CMR for assessment of LVEF is indicated to confirm an abnormal LVEF measured by TTE, when TTE images are non-diagnostic, or when the patient cannot tolerate a TTE [87].

Myocardial tissue characterization by CMR guides decision-making on further cardiotoxic therapeutic strategies. CMR can identify preexisting unrecognized myocardial infarctions as well as non-ischemic scar patterns, such as the sub-epicardial linear LGE pattern seen in patients with trastuzumab cardio-toxicity [88]. In some circumstances, stress perfusion CMR may aid in excluding underlying ischemia as the etiology of the cardiomyopathic process [89].

Further advanced cardiac imaging to detect early cardiac dysfunction in women receiving breast cancer therapy is on the horizon. Myocardial strain measured by CMR is clinically feasible [90] and holds promise for monitoring of cardio-toxicity, as determined in one study where strain decreased after low to moderate anthracycline-based therapy (− 17.7 ± 0.4% to − 15.1 ± 0.4%; *p* = 0.0003) [91]. Other reports [92, 93] suggest that CMR can detect a reduction of LV mass early after anthracycline-based chemotherapy, which was associated with worsening heart failure symptoms, independently of LVEF.

Novel CMR tools such as ECV and native T1 mapping can detect abnormality in the myocardial interstitial spaces after anthracycline exposure as compared to pretreatment values and cancer-free controls (ECV: 30.4 ± 0.7% vs 27.8 ± 0.7% and 26.9 ± 0.2%, respectively, *P* < 0.01) [94, 95].

#### Cardiac involvement in autoimmune and rheumatic disease

##### Rheumatoid arthritis

**Fig. 4:**
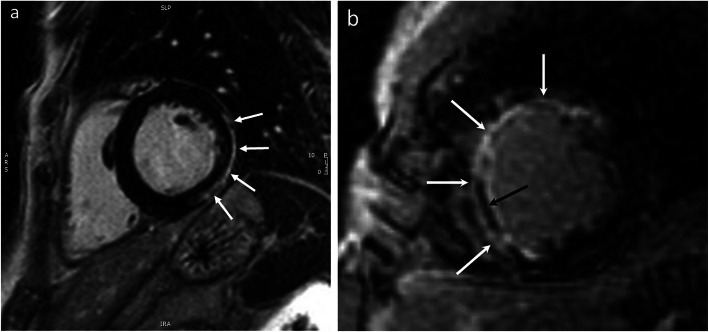
Rheumatoid arthritis and cardiac injury. Short-axis LGE image in 2 patients with rheumatoid arthritis: the left panel shows epicardial LGE of the basal inferolateral wall (**a** white arrows), due to myocarditis; the right panel shows near transmural anteroseptal myocardial infarction (**b** white arrows) with areas of microvascular obstruction (MVO, black arrow), due to left anterior descending coronary artery occlusion

Rheumatoid arthritis is a multi-system inflammatory disorder affecting 1% of the population, and is 3 times more frequent in women [96]. This condition can be associated with severe cardiovascular disease that contributes to reduction in life expectancy, especially in patients who are sero-positive for rheumatoid factor [97]. Heart disease in rheumatoid arthritis can present in various forms including: 1) inflammatory reactions of the pericardium, myocardium, and/or endocardium [98] (Fig. 4a), 2) coronary artery disease as ACS, acute MI, or as coronary microvascular dysfunction [99] (Fig. 4b), 3) heart failure due to inflammatory, valvular, or ischemic causes [100], and 4) amyloidosis and restrictive cardiomyopathy [100].

Currently, echocardiography, SPECT, CCT and CMR are used to evaluate the presence and extent of cardiovascular disease in rheumatoid arthritis patients. The advantage of CMR in patients with rheumatoid arthritis is that it is the only currently available non-invasive test that can directly visualize the extent of myocardial involvement in the various forms as mentioned above [101].

##### Systemic sclerosis

Systemic sclerosis is an autoimmune connective tissue disorder that mainly affects women, characterized by vascular dysfunction and multi-organ fibrosis [102, 103]. The heart is commonly involved [102], and the pericardium can also be affected [104]. Direct cardiac involvement may be seen in the form of cardiac fibrosis, myocarditis, dilated cardiomyopathy, heart failure, premature CAD, conduction system abnormalities, and valvular disease. Indirect cardiac involvement can also develop as sequela of pulmonary hypertension (PH) and renal crisis. Cardiovascular disease can remain subclinical, but systemic sclerosis patients with cardiovascular clinical features are at greater risk of deterioration and premature cardiovascular death [105], highlighting the importance of early detection and monitoring of myocardial and vascular involvement in all systemic sclerosis patients [106].

Currently, TTE is the cornerstone investigation for cardiac function, valve morphology, and pulmonary pressure assessment in this population. However, CMR can demonstrate early cardiac abnormalities before cardiac dysfunction. LGE imaging with CMR in systemic sclerosis patients may show evidence of focal fibrosis with a non-ischemic pattern. In the largest CMR study of systemic sclerosis to date, Hachulla et al. found evidence of focal fibrosis in 21% of patients [107]. In the same cohort, CMR detected findings suggestive of myocardial edema in 12% of patients presenting with high signal intensity ratio on T2-weighted imaging [107]. Other authors have reported even higher prevalence of focal LGE in systemic sclerosis patients approaching 43 to 66% [102, 108–110]. In systemic sclerosis patients with LGE (fibrosis or infarction), LV and RV strain have been found to be impaired (− 18 to − 17% for LV and − 22% for RV) compared to systemic sclerosis patients without LGE (− 20% for LV and − 27% for RV) [111], highlighting the potential role of CMR for detection of early cardiac dysfunction. In systemic sclerosis patients with preserved global ventricular function, Thuny et al. demonstrated impairment in peak LV systolic circumferential strain and peak LV diastolic strain rate by CMR [102].

Other advanced CMR techniques can also detect abnormal myocardial tissue characteristics in systemic sclerosis, including T1 mapping and ECV quantification. Ntusi et al. demonstrated significantly higher native myocardial T1 values in systemic sclerosis patients compared to controls [102]. The same investigators were able to detect a larger area of abnormal myocardial native T1 values (> 990 ms) and expansion of ECV beyond the boundaries of myocardial edema on T2-weighted imaging of systemic sclerosis patients, suggesting a combination of low-grade inflammation and increased interstitial volume [102]. The abnormal native T1 and ECV values were associated with worse disease activity and severity [102].

In addition to abnormalities in myocardial tissue characteristics, evidence of microvascular dysfunction has been demonstrated with adenosine stress perfusion CMR in systemic sclerosis patients, with the identification of mostly non-segmental perfusion defects. Kobayashi et al. reported 56% of patients with systemic sclerosis had stress perfusion defects that did not necessarily match the focal fibrosis on LGE [112]. In another study, all systemic sclerosis patients had non-segmental perfusion defects, which were most commonly seen in those with Raynaud’s phenomenon and digital ulceration [113]. Finally, perfusion defects in asymptomatic systemic sclerosis patients have also been found to correlate with impaired strain [114].

##### Systemic lupus erythematosus

Systemic lupus erythematosus (SLE) is a chronic, relapsing and remitting, multisystem inflammatory disorder, occurring 8 to 15 times more commonly in women [115]. Cardiovascular disease is relatively common in SLE, up to 9 times compared to healthy members of the population [116], and many patients have subclinical cardiovascular involvement [117]. Pericarditis, myocarditis, and valve involvement are frequently seen, but most of the excess mortality is due to accelerated atherosclerosis and CAD [116, 118, 119] and lupus coronary arteritis can occur [120]. The rate ratio for MI in women with SLE aged 35 to 44 years is 52 times that of a comparative healthy population in the Framingham cohort [121]. SLE is characterized by several vascular processes, namely inflammation, Raynaud’s phenomenon, and a propensity to vascular thrombosis associated with antiphospholipid antibodies, typically in the absence of traditional cardiovascular risk factors [122].

Advanced CMR methods can detect silent myocardial involvement in SLE [123], offering the potential to improve risk stratification and monitor disease progression beyond or in supplement to assessment by echocardiography. Stress perfusion CMR has demonstrated evidence of inducible myocardial ischemia in 44% of subjects with SLE in the absence of obstructive CAD [124]. Myocardial necrosis and fibrosis have been demonstrated by CMR in SLE, with both ischemic and non-ischemic patterns of injury [125–127]. Evidence of active myocarditis has been demonstrated in SLE using T2-weighted imaging [128]. Additional recent evidence suggests that patients with SLE exhibit an increased native T1 and ECV and impaired strain [125], the latter associated with increased arterial stiffness. Finally, impaired myocardial energetics in lupus and rheumatoid arthritis on phosphorous CMR spectroscopy correlated with presence of LGE, myocardial perfusion abnormalities, LA size, ECV and native T1 [129]. Coronary vessel wall imaging by contrast enhanced CMR can detect subclinical enhancement of the coronary vessel wall, a potential novel direct marker of vessel wall injury and remodeling in patients with lupus coronary arteritis [130].

##### Vasculitis

Primary vasculitis is more prevalent in the female population and may be associated with episodic myocardial inflammation, accelerated atherosclerosis, and premature CAD [131]. CMR angiography (CMRA) provides a broad overview of the potential vascular abnormalities in these diseases, including detection and morphologic characterization of aneurysms, aortic valve involvement, coronaries, and branch vessels (subclavian, renal, iliac). CMR can readily detect additional abnormalities of great importance in large vessel vasculitis like Takayasu arteritis, including thrombus, dissection, stenosis of aorta and proximal vessels, vascular inflammation, and pericardial effusions [132]. In addition, inflammatory, stenotic, or occlusive lesions in the aorta, pulmonary arteries, subclavian, or other peripheral arteries detected by CMRA have been shown to correlate with disease activity [133].

Myocardial injury can be demonstrated using CMR in patients with vasculitis that preferentially involve the heart such as Churg-Strauss syndrome. In a small series of patients with this syndrome and a normal TTE, CMR showed impairment of LV function in about half of the patients, myocardial edema by T2 imaging in a third, and LGE in more than 80% [134]. A pattern of subendocardial LGE has been described in these patients [135].

#### Duchenne and Becker muscular dystrophies

**Fig. 5:**
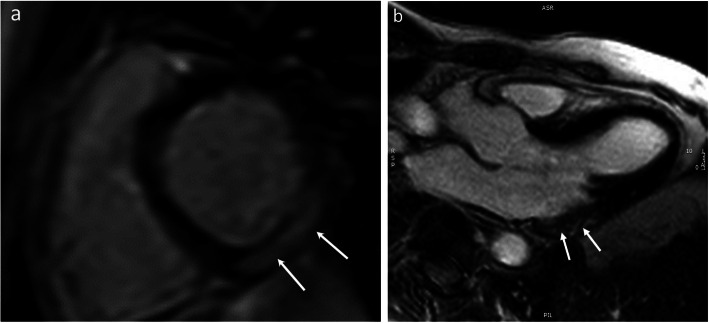
Duchenne muscular dystrophy. A woman with dystrophin mutation carrier status was screened for myocardial disease with CMR. Late gadolinium enhancement images demonstrated epicardial enhancement of the basal lateral wall in both the short-axis (**a**) and long-axis views (white arrows), a typical subtle pattern of myocardial injury seen in Duchenne muscular dystrophy

Duchenne and Becker muscular dystrophies result from mutations in the gene encoding for dystrophin. Female carriers of this X-linked recessive disorder also carry a risk of random X-inactivation that may leave cardiomyocytes with only the abnormal copy, fostering the understanding that these patients may also develop cardiomyopathy [136]. CMR demonstrates a high prevalence of myocardial disease in these patients with nearly half of serially screened female carriers showing either LV dysfunction (14%) or LGE abnormality (44%) in a recent series [137]. The lateral wall epicardial damage identified by LGE mirrors that seen in affected men (Fig. 5), underscoring the genetic mechanism of myocardial disease in these women. Given the high sensitivity of CMR in detecting often subclinical cardiac involvement in female carriers of dystrophin mutations, CMR studies that evaluate the long-term utility of initiating cardioprotective therapy in females are needed, as this is now considered standard of care for men with dystrophinopathies [138].

#### Women carriers of rare diseases

Fabry Disease is an X-linked lysosomal storage disorder caused by deficiency of the enzyme alpha-galactosidase A, and female carriers have significant cardiac involvement [139, 140]. In the United States, there is an estimated prevalence of 1 in 40,000 to 60,000 males affected by Fabry Disease according to the National Institutes of Health (https://www.fabrydisease.org, https://www.fabrydisease.org/index.php/about-fabry-disease/how-many-people-have-fabry-disease), but the number of affected female carriers is less understood.

Irrespective of sex, CMR can identify a pre-hypertrophic phenotype in Fabry Disease consisting of both sphingolipid deposits within the myocardium (detect by T1 mapping) and cardiac functional changes [141]. CMR is ideally suited to detect intramyocardial sphingolipid deposits with T1 mapping [142]; LGE and morphological abnormalities are also readily demonstrated.

Global longitudinal strain in Fabry Disease correlates with increased LV mass and presence of electrocardiogram (ECG) abnormalities [143]. In the LV hypertrophy-negative patients, global longitudinal strain is associated with a reduction in T1 mapping, consistent with sphingolipid deposition [143], which can potentially detect early disease in female carriers.

## Pulmonary arterial hypertension

Pulmonary hypertension (PH) is characterized by sustained elevation of pulmonary resistance with high mortality rate due to right heart failure [144]. Pulmonary arterial hypertension (PAH) is a rare disease, with manifest precapillary PH characterized by a resting mean pulmonary artery pressure ≥ 25 mmHg, in the presence of LA pressure ≤ 15 mmHg, and with preserved or reduced cardiac output. Survival rates are 67–73% after 3 years [145–147].

**Fig. 6:**
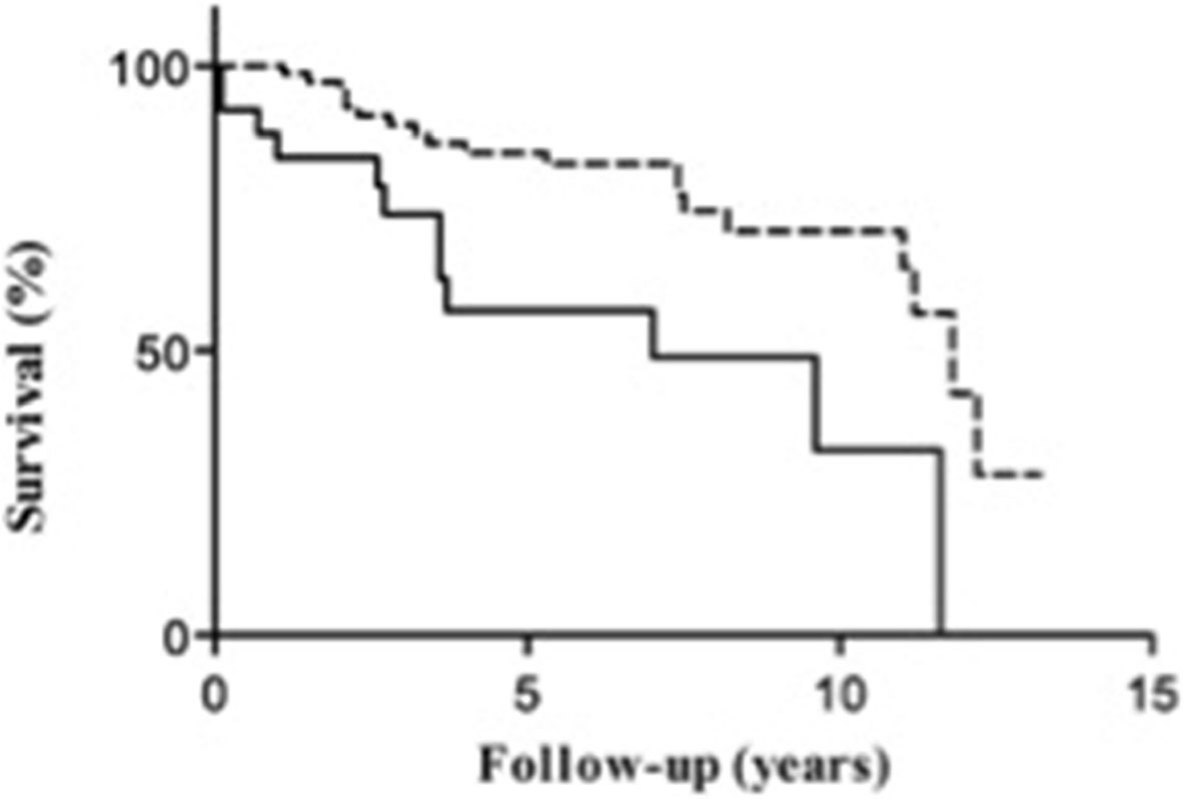
Sex differences in transplantation-free survival in pulmonary arterial hypertension. Transplant-free survival in male (solid line) and female (dashed line) patients with pulmonary arterial hypertension starting first-line pulmonary arterial hypertension-specific therapies (*P* = 0.002) [150]. Reprinted from CHEST, 145 [5], Jacobs W et al., The Right Ventricle Explains Sex Differences in Survival in Idiopathic Pulmonary Arterial Hypertension, 1230–1236. Copyright (2014), with permission from Elsevier

PAH is more common in women, with a particularly high female predominance in patients with PAH secondary to systemic sclerosis, where women constitute more than 80% of the population [144, 148]. CMR studies have shown that women with PAH have better RV function than men at baseline [149], and show greater improvement in RVEF following initiation of PAH-targeted medical treatment [150] compared to men (Fig. 6).

**Fig. 7:**
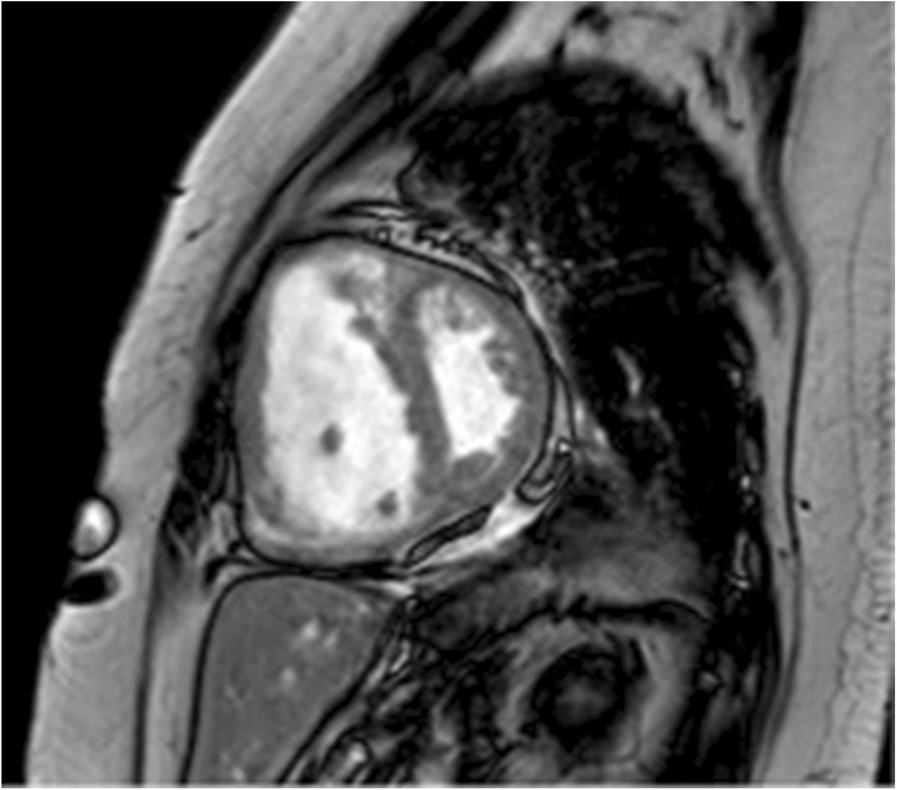
Pulmonary arterial hypertension. Short axis balanced steady state free precession cine image in patient with precapillary pulmonary hypertension in diastole demonstrating right ventricular dilatation and hypertrophy as well as bulging of the interventricular septum into the small left ventricle as a sign of high pulmonary arterial pressure. The septal flattening can easily be demonstrated by echocardiography, but the biventricular mass, volumetric, and functional quantification using CMR is superior to echocardiographic estimates

CMR is a reliable method to evaluate cardiac structure and function in PAH patients (Fig. 7), and can be used to predict prognosis [151, 152]. CMR-derived estimation of mean pulmonary arterial pressure has been suggested using septal angle and ventricular mass [153]. Early and reliable detection of ventricular dysfunction is important in patients with PH, and CMR has unique capabilities to quantify RV dysfunction and ventricular septal abnormalities [154]. Fibrosis at the RV insertions on the interventricular septum has been shown by LGE CMR technique in PH [155]. Of note, presence of LGE is related to the degree of RV dysfunction, severity of PH [155, 156], and poorer clinical outcomes [152] in these patients. RA volume measured by CMR can also predict clinical outcomes in PH patients. After multivariate adjustment for RVEF, increased RA volume was still associated with worse clinical outcome in a study of patients with pre-capillary PH, with a lung-transplantation or death hazard ratio of 2.1 (95%CI: 1.1–4.0) [157].

Several CMR biomarkers have been shown to predict mortality in idiopathic PAH, such as RV dilation, smaller RV stroke volume, low RVEF, and impaired LV filling [151]. Deterioration in these variables at follow up are the strongest predictors of poor outcome after 1 year [151]. The EURO-MR Study suggested that CMR could be used to assess clinical benefit of PAH-targeted medical therapy, where improvement of RV and LV function and volumes was associated with patient survival [158].

## Turner syndrome

Turner Syndrome is a genetic disorder affecting 1 in 2500 live female births, characterized by short stature, gonadal dysgenesis, as well as renal and cardiovascular anomalies [159]. Cardiovascular anomalies are present in at least 50% of women with Turner Syndrome [160], and there is an approximately 3-fold increase in age-related risk of mortality primarily due to cardiovascular abnormalities and atherosclerosis [161]. Currently, there is lack of a standardized cardiovascular risk assessment in Turner Syndrome. However, early detection of cardiovascular disorders is critical for initiation of appropriate therapies, and CMR has an expanding role in this population [162].

**Fig. 8:**
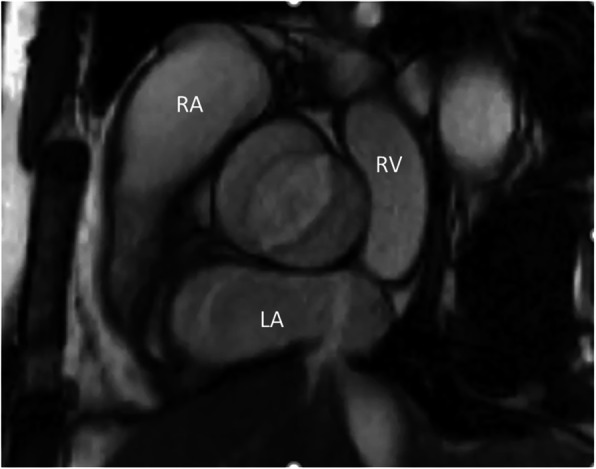
Bicuspid valve. Balanced steady-state, free precession cine CMR in the short-axis plane demonstrating a bicuspid aortic valve in a young woman with Turner syndrome. Echocardiography is first-line modality for assessment of cardiac valves. However, CMR can corroborate the valve morphology in case of suboptimal image quality with echocardiography. RA, right atrium; LA, left atrium; RV, right ventricle

The most common cardiovascular anomalies in Turner Syndrome include bicuspid aortic valve , aortic dilation, coarctation of the aorta, and anomalous pulmonary venous return [163]. Bicuspid aortic valve (Fig. 8) occurs in up to 30% of women with Turner Syndrome, and has been shown to be associated with an accelerated aortopathy in these patients [164].

CMR is an excellent tool to identify and monitor progression of Turner Syndrome abnormalities [164]. Hjerrild et al. reported CMR findings in 102 women with Turner Syndrome and found aortic diameter assessed by CMR correlated with age, blood pressure, bicuspid aortic valve, and a history of aortic coarctation [165]. Aortic dilation is present in 40% of women with Turner Syndrome, and the use of standard absolute values for aortic diameters in these women is inaccurate, owing to their body size. A more appropriate CMR parameter is ascending aortic size indexed to the body surface area, as 25% of the women with absolute values above 3.5 cm and 33% of the women with indexed values > 2.5 cm/m^2^ are subject to aortic dissection (6- to 100 fold higher) within 3 years of follow-up [166]. Women with Turner Syndrome have an increased risk of aortic dissection, and dissection occurs at a much earlier age than in the general population (30.4 years vs. 77 years) [167, 168]. In addition to assessment of aortic abnormalities, CMR can confirm the presence of anomalous pulmonary venous drainage, which is found in 10–15% of Turner Syndrome cases and can be particularly challenging to diagnosis in adults by TTE [169].

Cardiovascular abnormalities in Turner Syndrome patients may be under-diagnosed in childhood in the absence of screening, as shown in a study of 150 women with Turner Syndrome, where more than 40% of the subjects were found to have unknown cardiac anomalies [170]. As a result, CMR is recommended for screening in all children with Turner Syndrome, regardless of whether any cardiac anomalies have been detected by TTE; however, optimal timing of imaging is unclear. In general, it is recommended that CMR be performed at an age when sedation is not needed [171].

## CMR in pregnancy

CMR is a well-established method for imaging cardiovascular disease in pregnant women with potentially life-threatening abnormalities that cannot be completely characterized by TTE [172, 173]. As such, CMR can identify and characterize the severity of cardiovascular conditions that impose a significant risk for mother and offspring, and for which pregnancy is not recommended. Such conditions include Marfan Syndrome with significantly dilated aortic root, complex congenital heart disease (CHD), as well as severe left heart obstructive lesions and LV dysfunction [174]. The main role of CMR in pregnancy is risk stratification to inform the most suitable mode of delivery, to plan adequate cardiovascular care during delivery and postpartum, and to assist in recommendation for pregnancy interruption only when indicated.

**Fig. 9:**
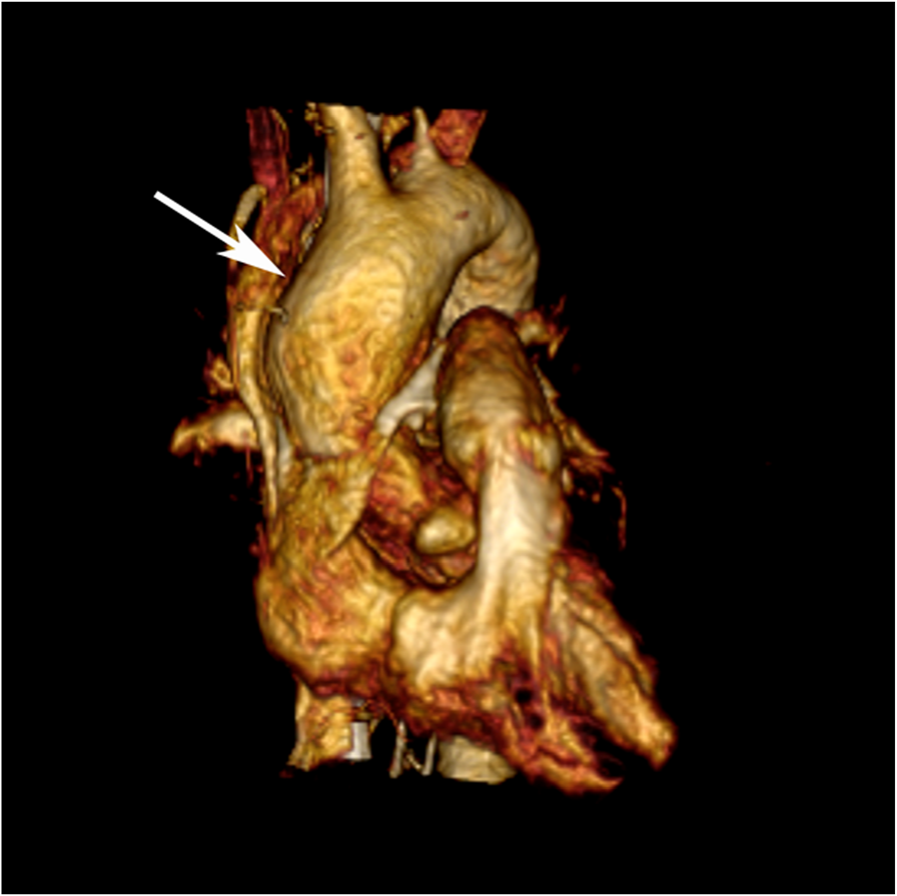
Dilated ascending aorta in pregnant patient. 3D volume rendering reformation of a non-contrast CMR angiogram in a patient with bicuspid aortic valve and Marfan Syndrome shows dilatation of the ascending aorta (arrow). In this patient with a maximum ascending aortic dimension of 45 mm, close clinical monitoring was recommended during pregnancy and delivery

The most common indications for CMR during pregnancy are suspected aortic dissection, aortic aneurysm, aortic coarctation, cardiomyopathy/myocarditis, and postoperative complex CHD. While aortic dissection is a rare event during pregnancy, it is associated with up to 10% mortality rate [175]. If dissection occurs, it is most frequently during the third trimester or the postpartum period. Patients with bicuspid aortic valve, aortic coarctation, and collagen vascular diseases have increased risk of aortic dissection [176]. Therefore, it has been suggested that a dilated aorta with a maximum diameter of > 50 mm (Fig. 9) in bicuspid aortic valve patients, and > 45 mm in Marfan Syndrome patients, is a threshold for potential pregnancy interruption during the first trimester [176]. Any woman with Marfan Syndrome presenting with chest or intrascapular pain during pregnancy should have urgent cross-sectional imaging of the aorta to exclude dissection, and this should preferably be with CMR [174].

In pregnant women with postoperative CHD, CMR evaluation should be optimized for quantification of ventricular volumes and function, functionality of conduits, baffles and grafts, as well as assessment of the pulmonary and aortic valves [174, 176]. Of note, high maternal and fetal mortality rates occur with a LVEF < 40%, dilated and dysfunctional systemic RV, as well as pulmonary or aortic valve obstructive lesions [177, 178]. Women with a systemic RV are at particular risk of maternal pregnancy related complications, and a recent analysis of 17 women with transposition of the great arteries who had undergone atrial switch surgery revealed that all pregnancy related cardiac complications occurred in women with a systemic RVEF < 35% [179].

Many women with CHD undergo serial imaging by CMR for cardiovascular surveillance and risk stratification prior or after pregnancy [180, 181]. CMR may be particularly useful in women with moderate or severe forms of CHD for risk stratification. In 28 women with aortic coarctation (4 native, 24 repaired) who underwent CMR within 2 years of pregnancy, a minimum aortic diameter ≤ 12 mm was identified as an important anatomic determinant of adverse cardiovascular outcomes. For each decrease in absolute aortic diameter of 1 mm, or indexed aortic diameter of 1 mm/m^2^, there was a three-fold increase in odds of occurrence of a cardiovascular event during pregnancy [180]. CMR has also been used to determine the degree of RV remodeling following pregnancy in women with repaired tetralogy of Fallot (TOF). Egidy-Assenza et al. compared data from sequential CMRs from 13 women with repaired TOF who completed pregnancy and from a matched comparison group of 26 nulliparous women with repaired TOF. The rate of increase of indexed RVEDV in the pregnancy group was higher than the comparison group (4.1 ± 1.1 ml/m^2^/year vs. 1.6 ± 0.6 ml/m^2^/year, *p* = 0.07) [181].

According to CMR safety guidelines, there are no reports of clinical CMR during pregnancy inducing deleterious effects to mother or fetus [182]. Indeed, a recent large cohort study demonstrated that exposure to MRI during the first trimester of pregnancy compared with non-exposure was not associated with increased risk of harm to the fetus or in early childhood [183]. However, the same study showed that exposure of gadolinium-based contrast agent (GBCA) at any time during pregnancy was associated with an increased risk of a broad set of rheumatological, inflammatory, or infiltrative skin conditions and risk of stillbirth or neonatal death [183]. Accordingly, the American College of Radiology does not recommend GBCA administration during pregnancy based on the absence of sufficient evidence to conclude no risk, unless the benefits significantly outweigh the risks to mother and fetus [182]. GBCA should only be administered for CMR examination after non-contrast techniques have been attempted and failed to answer the clinical question.

## Conclusions

CMR is high-spatial/temporal resolution, non-invasive, non-ionizing radiation imaging modality that adds value in the identification and prognostication of cardiovascular diseases in both sexes, with unique advantages in women. CMR is particularly suitable to identify early cardiovascular disease by means of myocardial characterization and cardiac functional assessment without the use of radiation. In women with chest pain, CMR is unique in precisely identifying ischemia in the absence of obstructive coronary lesion and in establishing alternate diagnoses for MINOCA. CMR is also useful in early detection, severity assessment and monitoring of cardiac diseases specific to women, such as peripartum cardiomyopathy, chemotherapy induced cardiomyopathy after breast cancer treatment, PAH, rheumatological conditions affecting the heart, and Takotsubo cardiomyopathy.

Finally, in CHD and pregnancy related issues, CMR also provides added benefits compared to other non-invasive imaging modalities. CMR is an excellent tool to evaluate cardiovascular anatomy, function, and pathology in women with cardiovascular diseases.

## Data Availability

Data sharing is not applicable to this article as no datasets were generated or analyzed during the current study.

## Abbreviations

ACS: Acute coronary syndromes
AHA: American Heart Association
CAD: Coronary artery disease
CCT: Cardiac computed tomography
CHD: Congenital heart disease
CMR: Cardiovascular magnetic resonance
CMRA: Cardiovascular magnetic resonance angiography
ECG: Electrocardiogram
EF: Ejection fraction
FFR: Fractional flow reserve
GBCA: Gadolinium based contrast agent
LA: Left atrium/left atrial
LAV: Left atrial volume
LAVI: LLeft atrial volume index
LGE: Late gadolinium enhancement
LV: Left ventricle/left ventricular
LVEDV: Left ventricular volume
LVEDVI: Left ventricular volume index
LVEF: Left ventricular ejection fraction
LVESV: Left ventricular end-systolic volume
LVESVI: Left ventricular end-systolic volume index
LVM: Left ventricular mass
LVMI: Left ventricular mass index
LVSV: Left ventricular stroke volume
LVSVI: Left ventricular stroke volume index
MI: Myocardial infarction
MINOCA: Myocardial infarction in non-obstructed coronary arteries
MRA: Magnetic resonance angiography
MPRI: Myocardial perfusion reserve index
MVO: Microvascular obstruction
NSTEMI: Non ST-elevation myocardial infarction
PPCM: Peripartum cardiomyopathy
PAH: Pulmonary arterial hypertension
PH: Pulmonary hypertension
RA: Right atrium/right atrial
RAV: Right atrial volume
RAVI: Right atrial volume index
RV: Right ventricle/right ventricular
RVEDV: Right ventricular end-diastolic volume
RVEDVI: Right ventricular end-diastolic volume index
RVEF: Right ventricular ejection fraction
RVESV: Right ventricular end-systolic volume
RVESVI: Right ventricular end-systolic volume index
SLE: Systemic lupus erythematosus
SPECT: Single-photon emission computed tomography
STEMI: ST-elevationmyocardial infarction
TCM: Takotsubo cardiomyopathy
TOF: Tetralogy of Fallot
TTE: Transthoracic echocardiography
